# Bis(tricyclic) Aromatic Enes That Exhibit Efficient Fluorescence in the Solid State

**DOI:** 10.3390/molecules29225361

**Published:** 2024-11-14

**Authors:** Masaki Shimizu, Kenta Nishimura, Mizuki Mineyama, Rin Terao, Tsuneaki Sakurai, Hiroshi Sakaguchi

**Affiliations:** 1 Faculty of Molecular Chemistry and Engineering, Kyoto Institute of Technology, 1 Hashikami-cho, Matsugasaki, Sakyo-ku, Kyoto 606-8585, Japan; 2 Institute of Advanced Energy, Kyoto University, Gokasho, Uji 611-0011, Japan

**Keywords:** acridine, charge transfer, crystal, fluorescence, strained alkenes, thioxanthene, xanthene

## Abstract

We report herein that bis(tricyclic) aromatic enes (BAEs) consisting of 6-6-6-membered frameworks such as acridine, xanthene, thioxanthene, and thioxanthene-*S*,*S*-dioxide act as a new class of organic luminophores that exhibit blue-to-green fluorescence in the solid state and in polymer film with good to excellent quantum yields. The BAEs were prepared by the palladium-catalyzed double cross-coupling reaction of phenazastannines or 10,10-dimethyl-10*H*-phenothiastannin with 9-(dibromomethylene)xanthene, 9-(dibromomethylene)thioxanthene, or 9-(dibromomethylene)-9*H*-thioxanthene-10,10-dioxide. Microcrystals or powder samples of the BAEs exhibited brilliant fluorescence with good to high quantum yields (*Φ* = 0.45–0.88). Furthermore, more efficient emission of blue-to-green light (*Φ* = 0.59–0.91) was observed for the BAEs dispersed in the poly(methyl methacrylate) (PMMA) films. Density functional theory (DFT) calculations suggest that the photo-absorption of the (thio)xanthene moiety-containing BAEs proceeds via π–π* transitions, whereas the optical excitation of 10,10-dioxido-9*H*-thioxanthene moiety-containing BAEs involves an intramolecular charge transfer from the acridine/thioxanthene part to the electron-accepting 10,10-dioxido-9*H*-thioxanthene moiety.

## 1. Introduction

**Figure 1:**
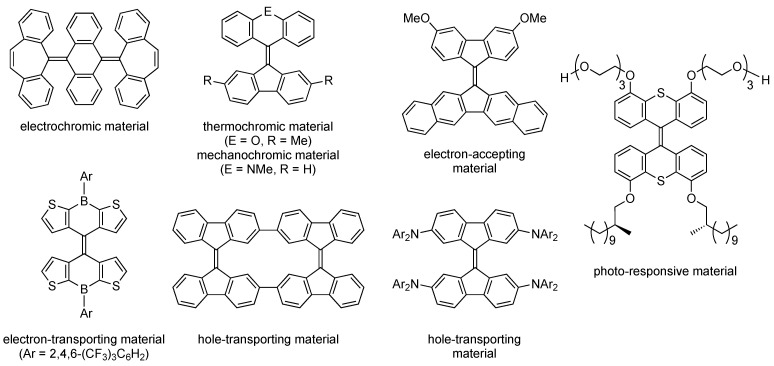
Examples of functional bis(tricyclic) aromatic enes (BAEs).

Bis(tricyclic) aromatic enes (BAEs) are attracting increasing interest as key frameworks for the development of functional organic materials. This is because BAEs exhibit unique electronic behavior and stimuli-responsiveness originating from their cross-conjugated electronic structures and the flexible conformations of the overcrowded alkene moieties. To date, several types of BAE-based functional materials have been developed that exhibit electrochromism [1,2], mechanochromism [3,4], thermochromism [5,6,7,8,9], charge transport [10,11,12,13,14,15], and photosensitivity [16,17,18,19,20,21,22] (Figure 1). However, there are few BAEs that exhibit efficient fluorescence, be that in a solution, in the solid state, or in a polymer film [23,24,25,26]. Organic luminophores that exhibit efficient solid-state emission are essential for the advancement of organic light-emitting diodes [27,28,29], organic light-emitting field-effect transistors [30,31,32,33], and organic solid-state lasers [34,35,36,37]. Furthermore, luminescent solar concentrators (LSCs) have received renewed attention in the field of renewable energy-generating devices [38,39,40,41,42,43,44]. Since LSCs consist of a transparent polymer waveguide, such as poly(methyl methacrylate) (PMMA), doped with a fluorophore, organic luminophores that exhibit efficient fluorophores in polymer film are very attractive as dopants for LSCs. This presents a significant opportunity to explore and develop highly fluorescent BAEs that efficiently fluoresce in the solid state and in a polymer film, expanding their potential as versatile multifunctional optoelectronic materials.

**Figure 2:**
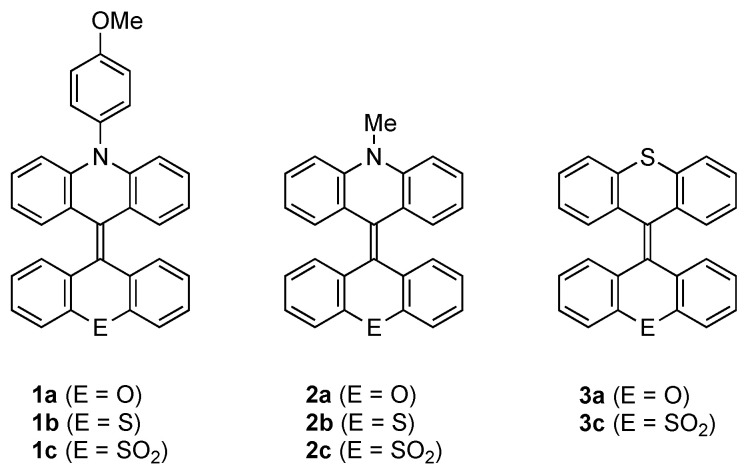
Molecular structures of heteromerous BAEs **1**–**3**.

We previously developed the palladium-catalyzed annulation of phenazastannines with 1,1-dibromoalkenes as a facile method for the synthesis of acridine moiety-containing heteromerous BAEs **1**–**3** (Figure 2) [45,46,47]. When we monitored the progress of the annulation reaction by thin-layer chromatography (TLC), we realized that the product spots on the TLC plates luminesced brilliantly on irradiation with a UV lamp (365 nm). This observation suggested that the synthesized BAEs **1**–**3** exhibit solid-state fluorescence and prompted us to investigate their luminescent properties. Herein, we report the single-crystal structures, absorption, and fluorescence properties and theoretical calculations of heteromerous BAEs **1**–**3** (Figure 2) and demonstrate that they are a promising new class of organic luminophores exhibiting efficient solid-state fluorescence in the blue-to-green region.

## 2. Results and Discussion

### 2.1. Synthesis

**Scheme 1:**
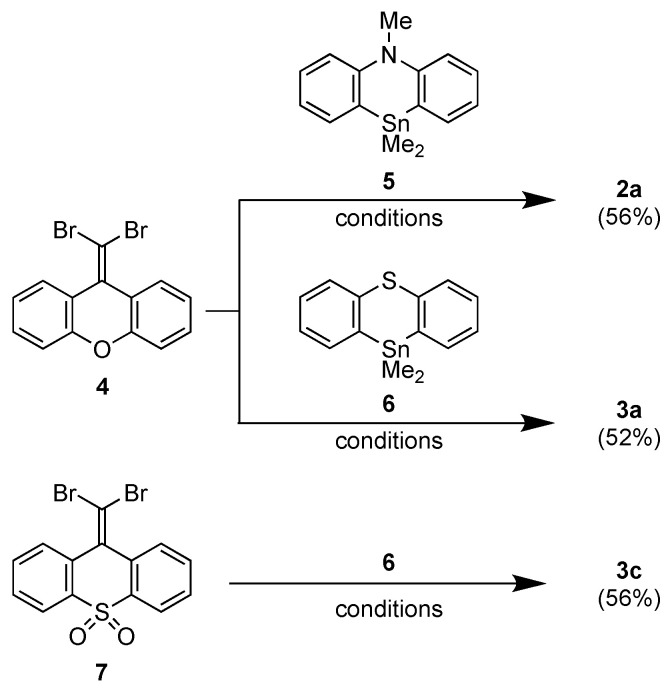
Synthesis of **2a**, **3a**, and **3c** (conditions: Pd_2_(dba)_3_ (5 mol%), PCy_3_ (20 mol%), CsF (10 eq), and 1,4-dioxane, 130 °C).

The preparation of acridine moiety-containing BAEs **1a**, **1b**, **1c**, **2b**, and **2c** has been reported previously and involves the Pd-catalyzed annulation of phenazastannines with the corresponding 1,1-dibromoalkenes [45]. Using the same protocol, **2a** and **3a** were synthesized in 56% and 52% yields from 9-(dibromomethylene)xanthene (**4**) [48] and 10,10-dimethyl-5-methylphenazastannine (**5**) [49] or 10,10-dimethyl-10*H*-phenothiastannin (**6**) [49], respectively (Scheme 1). The annulation of 9-(dibromomethylene)-9*H*-thioxanthene-10,10-dioxide (**7**) [45] with **6** afforded **3c** in a 56% yield. The decomposition temperatures (*T*_d_) of **1**–**3,** at which 5% of the mass was lost, were 291 (**1a**), 304 (**1b**), 322 (**1c**), 278 (**2a**), 282 (**2b**), 309 (**2c**), 256 (**3a**), and 303 °C (**3c**), indicating that the BAEs were thermally very stable.

### 2.2. Single-Crystal Structures

**Figure 3:**
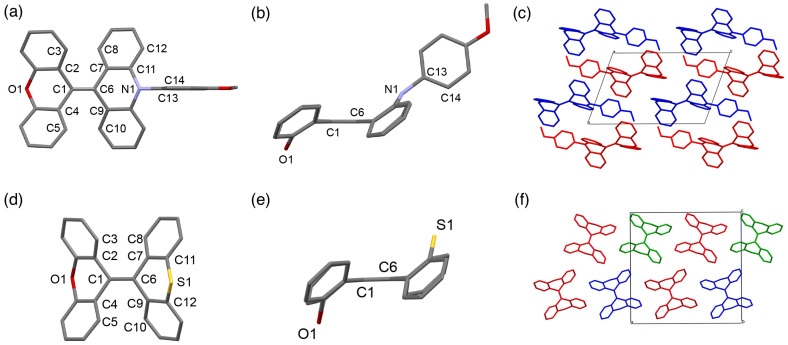
(**a**) Top view of the molecular structure of **1a**. (**b**) Side view of the molecular structure of **1a**. Hydrogen atoms are omitted for clarity. Selected bond lengths [Å], angles [°], and dihedral angles [°]: C1–C6 1.358 Å, ∠C2–C1–C6 124.92°, ∠C4–C1–C6 125.72°, ∠C2–C1–C4 109.36°, ∠C1–C6–C7 124.11°, ∠C1–C6–C9 125.12°, ∠C7–C6–C9 110.75°, ∠C2–C1–C6–C7 0.10°, ∠C4–C1–C6–C9 1.16°, and ∠C11–N1–C13–C14 79.06°. (**c**) Crystal structure of **1a** [space group: P-1; unit cell dimensions: *a* = 8.3291(9), *b* = 10.3629(12), *c* = 15.2598(17), α = 107.212(2), β = 94.588(2), and γ = 108.454(2)]. Each molecule in packing diagram is color-coded by symmetry equivalence. (**d**) Top view of the molecular structure of **3a**. (**e**) Side view of the molecular structure of **3a**. Hydrogen atoms are omitted for clarity. Selected bond lengths [Å] angles [°], and dihedral angles [°]: C1–C6 1.346 Å, ∠C2–C1–C6 125.37°, ∠C4–C1–C6 124.72°, ∠C2–C1–C4 109.88°, ∠C1–C6–C7 124.05°, ∠C1–C6–C9 123.58°, ∠C7–C6–C9 112.36°, ∠C2–C1–C6–C7 3.16°, and ∠C4–C1–C6–C9 0.05°. (**f**) Crystal structure of **3a** [space group: Pca21; unit cell dimensions: *a* = 18.282(2), *b* = 5.4902(7), and *c* = 18.355(2)]. Each molecule in the packing diagram is color-coded by symmetry equivalence.

Single crystals of **1a** and **3a** suitable for X-ray diffraction analysis were obtained by recrystallization from a solution of ethyl acetate and dichloromethane, respectively. CCDC numbers of **1a** and **3a** are CCDC 2180600 and 2180603. Compound **1a** crystallized in the triclinic space group *P*-1. As shown in Figure 3a,b, the tetraarylated ethene moiety (C2(C4)C1=C6(C7)C9) was flat, having dihedral angles of 0.10° (∠C2–C1–C6–C7) and 1.16° (∠C4–C1–C6–C9). The length of the C1=C6 bond is 1.358 Å, slightly longer than a standard value of the C=C bond (1.34 Å) [50], and the ∠C2–C1–C6, ∠C4–C1–C6, ∠C2–C1–C4, ∠C1–C6–C7, ∠C1–C6–C9, and ∠C7–C6–C9 bond angles are 124.92°, 125.72°, 109.36°, 124.11°, 125.12°, and 110.75°, respectively, which are significantly deviated from the ideal *sp*^2^ carbon angle of 120°. The unusual bond angles probably reduced the steric crowding around the tetraarylated ethene moiety. The central six-membered rings of the acridine and xanthene moieties adopted a boat-like conformation. The methoxyphenyl group was almost perpendicular to the acridine moiety, having a dihedral angle of 79.06° (∠C11–N1–C13–C14), suggesting that the *N*-aryl group did not participate in the conjugated system of the acridine moiety. Probably due to the bent molecular framework, there is no close packing between the BAE cores (Figure 3c), which is an attractive characteristic for the realization of efficient solid-state emissions. Compound **3a** crystallized in the orthorhombic space group *Pca*2_1_. As observed for **1a**, the tetraarylated ethene moiety (C2(C4)C1=C6(C7)C9) of **3a** was also flat, having dihedral angles of 3.16° (∠C2–C1–C6–C7) and 0.05° (∠C4–C1–C6–C9), and the bond angles at the *sp*^2^ carbons differed significantly from 120° (125.37° for ∠C2–C1–C6, 124.72° for ∠C4–C1–C6, 109.88° for ∠C2–C1–C4, 124.05° for ∠C1–C6–C7, 123.58° for ∠C1–C6–C9, and 112.36° for ∠C7–C6–C9) (Figure 3d). The conformations of the pyran and thiopyran rings were characterized as boat conformations (Figure 3e). Each molecule in the crystal forms a loose packing structure, which is advantageous for avoiding concentration quenching in the solid state (Figure 3f).

### 2.3. Absorption Properties in Toluene

**Figure 4:**
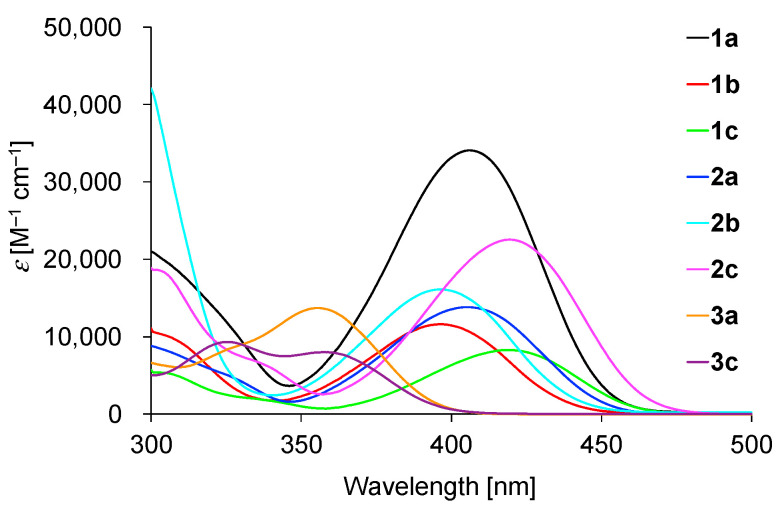
Absorption spectra of **1**–**3** in toluene.

The absorption data and spectra of **1**–**3** in toluene are shown in Table 1 and Figure 4, respectively. The absorption maxima of **1a**, **1b**, and **1c** are almost the same as those of **2a**, **2b**, and **2c**, respectively, indicating that the nitrogen substituent does not influence the energy gaps between the highest occupied molecular orbitals (HOMOs) and lowest unoccupied molecular orbitals (LUMOs). The maxima of **1a**–**1c** and **2a**–**2c** were red-shifted in the order **1b**, **1a**, **1c**, **2b**, **2a**, and **2c**. The maxima of **3a** and **3c** were significantly blue-shifted compared to those of **1** and **2**, which is reasonable considering that the electron-donating nature of a sulfur atom is less than that of a nitrogen atom.

### 2.4. Fluorescence Properties in Toluene

**Figure 5:**
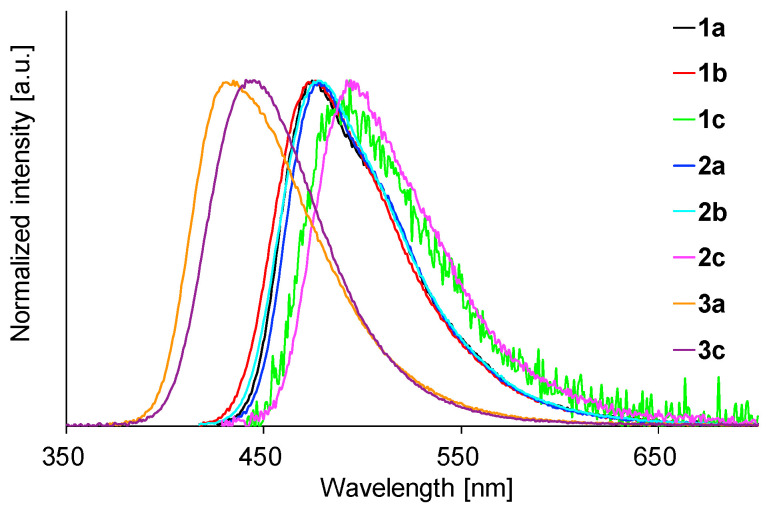
Fluorescence spectra of **1**–**3** in toluene.

Toluene solutions of **1**–**3** were faintly or moderately fluorescent (Table 2). The spectra are shown in Figure 5. The emission maxima of **1a**–**1c** and **2a**–**2c** appeared at 475–494 nm, whereas **3a** and **3c** fluoresced at 435 and 441 nm. The shorter wavelengths of the emission maxima of **3a** and **3c** compared to those of **1a**–**1c** and **2a**–**2c** are consistent with the trend in the absorption maxima. In contrast, the fluorescence maxima of **1a** and **2a** were almost the same as those of **1b** and **2b**, respectively, which is inconsistent with the absorption data. These results suggest that the electronic structures of the lowest singlet excited states (S_1_ states) of isolated **1a** and **1b** as well as **2a** and **2b** are quite similar (see Theoretical Calculations). Except for those of **1c** and **2c** (0.04 and 0.06, respectively), the quantum yields of **1**–**3** in toluene were moderate. Since the radiative decay constants (*k*_r_) of **1c** and **2c** were comparable to those of the others, the primary cause of the very low quantum yields of an SO_2_ moiety-containing **1c** and **2c** in toluene is the large nonradiative decay constants (*k*_nr_) of 32.0 and 23.5, respectively, which are an order of magnitude larger than those of the others (see Table S1 in Supplementary Material).

### 2.5. Fluorescence Properties in the Solid State

**Figure 6:**
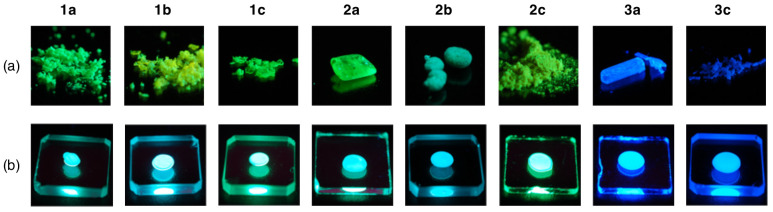
Fluorescence images of **1**–**3** in the solid state (**a**) and in PMMA films (**b**) upon irradiation with a UV lamp (365 nm).

**Figure 7:**
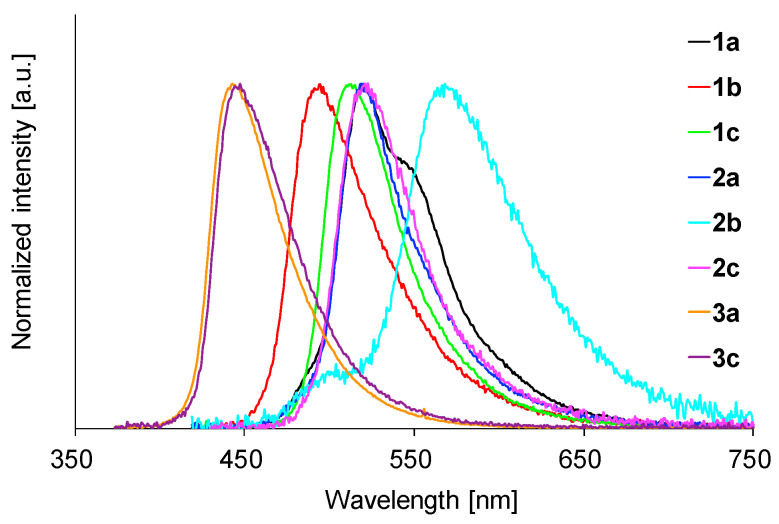
Fluorescence spectra of **1**–**3** in the solid state.

In sharp contrast to the weak fluorescence in toluene, brilliant green fluorescence (*λ*_em_ = 494–568 nm) was observed for the solid samples of **1** and **2**. In contrast, **3a** and **3c** efficiently emitted blue fluorescence at 443 and 448 nm, respectively, in microcrystalline solid form (Figure 6a). The fluorescence data and spectra are summarized in Table 2 and shown in Figure 7. The fluorescence quantum yields were good to excellent (*Φ* = 0.45–0.88) and were higher than those in toluene, except for that of **2b** (*Φ* = 0.30). Until now, only 0.308 and 0.347 have been reported as solid-state quantum yields of BAEs for 9-(9*H*-xanthen-9-ylidene)-9*H*-xanthene [23] and tetrabenzo[5.7]fulvalene [24]. Thus, these results demonstrate that the developed BAEs are a novel class of organic fluorophores that exhibit efficient fluorescence in the solid state [51,52,53,54,55,56,57]. The fluorescence maximum and quantum yield of **2b** were 568 nm and 0.30, respectively. Compared to other BAEs, the maximum was significantly red-shifted, and the yield was much lower, suggesting that **2b** was densely aggregated in the solid state, and thus, suffered from severe concentration quenching.

### 2.6. Fluorescence Properties in PMMA Film

**Figure 8:**
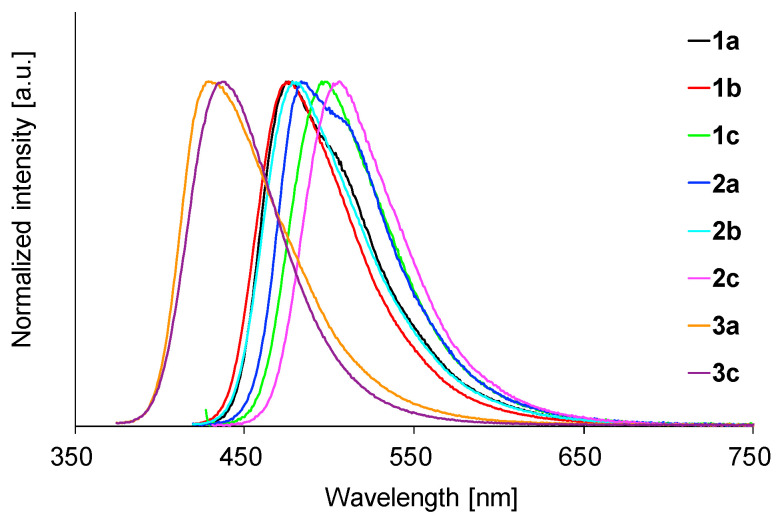
Fluorescence spectra of **1**–**3** in PMMA film.

We next investigated the fluorescence properties of the BAEs in polymer film to explore their potential as LSC-applicable emitters. First, four polymer films were prepared from PMMA, polystyrene, poly(bisphenol A carbonate), and poly(vinyl acetate) using **1b** and **2c** as the dopants. The fluorescence spectra were almost the same regardless of the host polymer (Figure S7 for **1b** and Figure S8 for **2c**). The highest fluorescence quantum yields (0.91 for **1b** and 0.80 for **2c**) were observed with the PMMA films (the data are shown in Table S2). Therefore, PMMA was chosen as the host polymer and the fluorescence properties of the other BAEs in PMMA were investigated. The fluorescence data and spectra are shown in Table 2 and Figure 8. The films efficiently emitted fluorescence at 429–506 nm, which were like those in the solution case, indicating that each molecule dispersed in the PMMA film was spatially and electronically isolated, as in the solution case. The fluorescence quantum yields in the PMMA film were high to excellent (*Φ* = 0.59–0.91), probably because of the isolated and rigid environment of each luminophore. Consequently, **1**–**3** are considered very attractive fluorophores for LSC applications.

### 2.7. Theoretical Calculations

**Figure 9:**
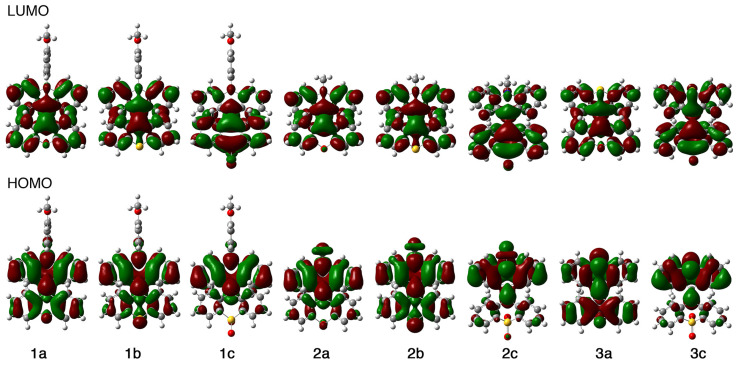
HOMO and LUMO drawings of **1**–**3**.

To gain insight into the electronic structures and photoluminescence mechanisms of **1**–**3**, density functional theory (DFT) calculations of the BAEs were carried out using the Gaussian 09 package [58]. Initially, DFT calculations were performed at the B3LYP/6-31+G(d) level of theory. However, the calculated HOMO–LUMO energy gaps did not correlate well with the experimentally estimated gaps from absorption spectra, suggesting that this functional and basis set combination was inadequate. In contrast, calculations at the B3LYP/cc-pVDZ level of theory yielded results that were consistent with the experimental data, with the exception of **2a**. Therefore, we used the B3LYP/cc-pVDZ levels of theory for theoretical calculation study. Initial structures for structural optimization were generated from the single-crystal structures of **1a** and **3a**. The calculated HOMO and LUMO energies (*E*_HOMO_ and *E*_LUMO_) and their energy gaps (Δ*E*_LUMO–HOMO_) are summarized in Table 3 along with the experimentally determined HOMO–LUMO gaps (Δ*E*_exp_), which were estimated from the absorption maxima in toluene. The magnitude of the relationship between the calculated HOMO–LUMO energy gaps is consistent with that of the experimentally estimated energy gaps except for that of **2a**. The discrepancy between the calculated and experimental HOMO-LUMO gaps for compound **2a** remains unclear. One possible explanation is the difference in phases: The calculated value is for the gas phase, while the experimental value is for the solution phase. The HOMOs of **1a**, **1b**, **2a**, **2b**, and **3a** were distributed over the entire BAE framework with a large contribution from the nitrogen and sulfur atoms despite their bent structures (Figure 9). The absence of the HOMOs over the methoxyphenyl group of **1a** and **1b** is probably due to the perpendicular orientation of the methoxyphenyl group to the BAE plane. The HOMOs of **1c**, **2c**, and **3c** containing an SO_2_ moiety are localized over the acridine skeleton and the central C=C bond, but not over the SO_2_ group nor over the two benzene rings bridged by the SO_2_ group. Meanwhile, the LUMOs of **1a**, **1b**, **2a**, and **2b** were delocalized over the BAE skeleton with little or no contribution from the sulfur or nitrogen atom. In the cases of **1c**, **2c**, and **3c**, which contain an SO_2_ moiety, the LUMOs were mostly accumulated over the 10,10-dioxido-9*H*-thioxanthene moiety. In short, these orbital distributions suggest that **1a**, **1b**, **2a**, **2b**, and **3a** are optically excited via the π–π* transitions of the BAE skeletons, while the photoexcitation of **1c**, **2c**, and **3c** occurs through a weak intramolecular charge transfer (ICT) from the acridine/thioxanthene moiety to the electron-accepting 10,10-dioxido-9*H*-thioxanthene moiety. The Lippert–Mataga plots of **1c** and **2c** were prepared with seven solvents (CHCl_3_, Et_2_O, EtOAc, THF, CH_2_Cl_2_, PhCN, and DMF) [59,60,61,62,63]. The plots fit the calibration lines defined by a linear approximation with moderate correlation coefficients of 0.701 and 0.731 (Figures S10 and S12), which experimentally supports ICT as the mechanism for the excitation process of the SO_2_-containing BAEs.

## 3. Materials and Methods

### 3.1. General

Melting and decomposition points were determined with a TG/DTA6200 Seiko Instrument (Seiko Instruments Inc., Chiba, Japan). ^1^H NMR spectra were recorded with a Varian Mercury 400 (400 MHz) spectrometer (Las Vegas, NV, USA). The chemical shifts of ^1^H NMR signals are expressed in parts per million, relative to the internal tetramethylsilane standard (*δ* = 0 ppm) or chloroform (*δ* = 7.26 ppm). Splitting patterns are indicated as follows: s, singlet; d, doublet; t, triplet; and m, multiplet. ^13^C NMR spectra were recorded with a Varian Mercury 400 (100 MHz) spectrometer with tetramethylsilane (*δ* = 0 ppm) or chloroform-*d* (*δ* = 77.0 ppm) as internal standard. Chemical shifts are given in parts per million, relative to the internal standards. IR attenuated total reflectance (IR-ATR) spectra were recorded by using a Shimadzu FTIR-8400 spectrometer (Kyoto, Japan) equipped with a Specac^®^ Quest ATR diamond accessory (Pleasantville, NY, USA). Fast atom bombardment high-resolution mass spectrometry (FAB–HRMS) analyses were performed with a JEOL JMS-700 spectrometer (Tokyo, Japan). TLC analyses were performed by using Merck Kieselgel 60 F254 silica plates (Rahway, NJ, USA). Silica gel column chromatography was carried out by using Merck Kieselgel 60 (230–400 mesh). 1,4-Dioxane was distilled from Na before use. All reactions were carried out under argon atmosphere.

### 3.2. Synthesis

#### 3.2.1. Synthesis of **2a**

In a glovebox, **5** (0.25 g, 0.75 mmol), **4** (0.18 g, 0.50 mmol), Pd_2_(dba)_3_ (23 mg, 0.025 mmol), PCy_3_ (28 mg, 0.10 mmol), CsF (0.76 g, 5.0 mmol), and 1,4-dioxane (25 mL) were added to a 50 mL vial. The vial was sealed, removed from the glovebox, and heated at 130 °C for 15 h. The reaction mixture was then filtered through a pad of Celite, and the filtrate was concentrated in vacuo. The crude product was purified using silica gel column chromatography (eluent: hexane/EtOAc 10:1) to give **2a** (0.10 g, 0.28 mmol, 56%) as a yellow solid. *T*_m_ (dec.) = 328 °C; TLC: *R*_f_ 0.40 (hexane/EtOAc 10:1); ^1^H NMR (CDCl_3_, 400 MHz): *δ* = 3.56 (s, 3H), 6.76–6.84 (m, 4H), 7.00 (d, *J* = 7.6 Hz, 2H), 7.09 (d, *J* = 7.6 Hz, 2H), 7.14–7.23 (m, 8H); ^13^C NMR (CDCl_3_, 100 MHz): *δ* = 33.4, 112.6, 116.9, 119.7, 120.2, 122.2, 123.8, 125.6 (2C), 127.6, 127.7, 128.0, 128.3, 144.8, 155.6; IR (neat): *ν* = 2958, 1591, 1550, 1494, 1452, 1433, 1386, 1338, 1288, 1265, 1161, 1134, 1120, 1058, 1028, 879, 844, 754, 742, 700 cm^−1^; HRMS (FAB) *m*/*z* [M^+^] calcd for C_27_H_19_NO: 373.1467, found: 373.1459.

#### 3.2.2. Synthesis of **3a**

The same procedure for the synthesis of **2a** was applied to **4** and **6**, affording **3a** in 52% yield as a colorless solid. *T*_m_ (dec.) = 295 °C; TLC: *R*_f_ 0.25 (hexane); ^1^H NMR (CDCl_3_, 400 MHz): *δ* = 6.77–6.78 (m, 4H), 7.01 (t, *J* = 7.6 Hz, 2H), 7.11 (d, *J* = 7.6 Hz, 2H), 7.15–7.23 (m, 6H), 7.58 (d, *J* = 7.6 Hz, 2H); ^13^C NMR (CDCl_3_, 100 MHz): *δ* = 116.7, 122.3, 123.9, 124.0, 126.0, 126.7, 127.8, 128.4, 128.8, 129.3, 130.3, 136.5, 136.7, 154.8; IR (neat): *ν* = 3913, 3709, 3616, 3564, 3047, 2349, 1579, 1556, 1467, 1435, 1394, 1373, 1238, 1228, 1195, 1095, 1062, 1026, 945, 883, 839, 790, 742, 727, 698 cm^−1^; HRMS (FAB) *m*/*z* [M^+^] calcd for C_26_H_16_OS: 376.0922, found: 376.0918.

#### 3.2.3. Synthesis of **3c**

The same procedure for the synthesis of **2a** was applied to **6** and **7**, affording **3c** in 56% yield as a colorless solid. *T*_m_ (dec.) = 366 °C; TLC: *R*_f_ 0.25 (hexane/EtOAc 10:1); ^1^H NMR (CDCl_3_, 400 MHz): *δ* = 6.89 (d, *J* = 7.6 Hz, 2H), 6.98 (t, *J* = 7.6 Hz, 2H), 7.07 (d, *J* = 7.6 Hz, 2H), 7.14–7.22 (m, 4H), 7.37 (t, *J* = 7.6 Hz, 2H), 7.59 (d, *J* = 7.6 Hz, 2H), 8.08 (d, *J* = 7.6 Hz, 2H); ^13^C NMR (CDCl_3_, 100 MHz): *δ* = 124.0, 126.1, 127.2, 127.5, 127.6 (2C), 130.2, 130.2, 130.8, 134.6, 135.2, 137.1, 138.7, 139.1; IR (neat): *ν* = 1454, 1437, 1311, 1294, 1267, 1165, 1141, 1116, 1066, 1049, 742, 723, 688 cm^−1^; HRMS (FAB) *m*/*z* [M^+^] calcd for C_26_H_16_O_2_S_2_: 424.0592, found: 424.0602.

### 3.3. X-Ray Crystallography of Single Crystals

Data were measured on a Bruker (Bremen, Germany) SMART APEX diffractometer (MoK*a* radiation, *l* = 0.71073 Å) at 300 K. The structures were solved by direct methods using SHELXTL programs (ver 6.1) and refined with full-matrix least-squares on *F*^2^. CCDC numbers of **1a** and **3a** are CCDC 2180600 and 2180603.

### 3.4. Preparation of Doped PMMA Films

The sample (1 mg) was dissolved in a CH_2_Cl_2_ (1 mL) solution of PMMA (99 mg). The resulting solution was dropped onto a quartz plate (10 mm × 10 mm) and spin-coated at 300 rpm for 40 s and then at 1000 rpm for 60 s. The deposited film was dried at room temperature in air for 12 h and under reduced pressure for 6 h.

### 3.5. Measurement of Absorption and Photoluminescence Spectra

The spectroscopic-grade toluene for photoluminescence measurements was purchased from Kanto Chemical Co., Inc. (Tokyo, Japan) and degassed with argon before use. The absorption spectra were obtained using a Shimadzu UV-2550 spectrometer. The fluorescence spectra and absolute quantum yields were recorded using a calibrated integrating sphere with a Hamamatsu Photonics C9920-02 Absolute PL Quantum Yield Measurement System (Hamamatsu, Japan), and the fluorescence lifetimes were measured with a Hamamatsu Photonics Quantaurus-Tau.

## 4. Conclusions

**Figure 10:**
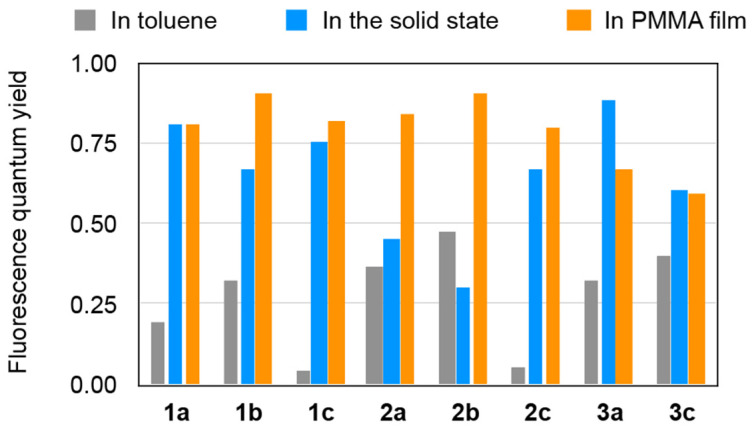
Graphical summary of the fluorescence quantum yields of **1**–**3**.

We demonstrated that heteromerous BAEs consisting of two different tricyclic moieties (in this study, acridine, xanthene, thioxanthene, or thioxanthene-*S*,*S*-dioxide) are thermally very stable, adopt bent conformations, and efficiently fluoresce in the solid state in the blue-to-green region. As summarized in Figure 10, the fluorescence quantum yields in the solid state, especially in PMMA film form, were good to excellent and significantly higher than those in the toluene solution. Given that to date there have been no reports of BAEs exhibiting solid-state emissions with high efficiency (*Φ* > 0.40), this study represents the first demonstration of BAEs that are highly luminescent in the solid state and add solid-state luminescent properties to the material functions exhibited by BAEs. We hope that the findings of this study will encourage materials chemists and scientists to use BAEs as solid-state fluorescent materials.

## Figures and Tables

**Table 1:** Absorption data of **1**–**3** in toluene.

Comp	*λ*_max_ (nm) *^a^*	*ε* (M^−1^ cm^−1^) *^b^*
**1a**	406	34,100
**1b**	396	11,600
**1c**	419	8300
**2a**	405	13,800
**2b**	396	16,100
**2c**	419	22,500
**3a**	356	13,700
**3c**	359	8000

*^a^ λ*_max_: absorption maximum. *^b^ ε*: molar absorption coefficients.

**Table 2:** Fluorescence data of **1**–**3** in toluene, in the solid state, and in PMMA film.

Comp	In Toluene ^*a*^			In the Solid State ^*b*^			In PMMA ^*c*^		
	*λ*_em_ *^d^*	*Φ ^e^*	*τ ^f^*	*λ*_em_ *^d^*	*Φ ^e^*	*τ ^f^*	*λ*_em_ *^d^*	*Φ ^e^*	*τ ^f^*
**1a**	475	0.20	1.3	520	0.81	5.92	478	0.81	5.49
**1b**	476	0.32	1.9	494	0.67	4.42	476	0.91	5.56
**1c**	493	0.04	0.3	513	0.76	4.95	496	0.82	4.87
**2a**	478	0.37	2.4	519	0.45	6.22	484	0.84	6.52
**2b**	478	0.48	3.2	568	0.30	6.49	478	0.91	5.98
**2c**	494	0.06	0.4	521	0.67	5.34	506	0.80	5.95
**3a**	435	0.32	1.8	443	0.88	4.66	429	0.67	4.02
**3c**	441	0.40	2.2	448	0.60	3.54	437	0.59	4.13

*^a^* Excitation wavelengths [nm]: 405 for **1a** and **2a**, 396 for **1b** and **2b**, 420 for **1c** and **2c**, 355 for **3a**, and 325 for **3c**. *^b^* Powder: **1a**, **1b**, **2b**, and **2c**; microcrystalline solid: **1c**, **2a**, **3a**, and **3c**. Excitation wavelength [nm]: 405 for **1a**, 396 for **1b**, 420 for **1c**, 405 for **2a**, 396 for **2b**, 420 for **2c**, 355 for **3a**, and 325 for **3c**. *^c^* Excitation wavelength [nm]: 405 for **1a**, 396 for **1b**, 380 for **1c**, 405 for **2a**, 396 for **2b**, 420 for **2c**, 355 for **3a**, and 325 for **3c**. *^d^ λ*_em_: fluorescence maximum [nm]. *^e^ Φ*: fluorescence quantum yield. *^f^ τ*: fluorescence lifetime [ns].

**Table 3:** HOMO and LUMO energies of **1**–**3** *^a^*.

Comp	*E*_HOMO_ [eV]	*E*_LUMO_ [eV]	Δ*E*_LUMO−HOMO_ [eV]	Δ*E*_exp_ [eV] *^b^*
**1a**	−4.96	−1.45	3.51	3.05
**1b**	−5.05	−1.40	3.65	3.13
**1c**	−5.45	−2.05	3.40	2.96
**2a**	−5.21	−1.47	3.74	3.06
**2b**	−5.10	−1.42	3.68	3.13
**2c**	−5.58	−2.15	3.43	2.96
**3a**	−5.54	−1.67	3.87	3.48
**3c**	−5.94	−2.07	3.87	3.45

*^a^* Calculated at the B3LYP/cc-pVDZ levels. *^b^* Δ*E*_exp_: HOMO–LUMO energy gaps estimated from the absorption maxima in toluene.

## Data Availability

The data presented in this study are available in the Supplementary Materials.

## Supplementary Materials

The following supporting information can be downloaded at: https://www.mdpi.com/article/10.3390/molecules29225361/s1, Figures S1–S6: ^1^H and ^13^C NMR spectra of **2a**, **3a**, and **3c**; Figure S7: Fluorescence spectra of **1b** in polymer film; Figure S8: Fluorescence spectra of **2c** in polymer film**;** Figure S9: Absorption and fluorescence spectra of **1c** in various solvents; Figure S10: Lippert–Mataga plots of **1c**; Figure S11: Absorption and fluorescence spectra of **2c** in various solvents; Figure S12: Lippert–Mataga plots of **2c**; Table S1: Fluorescence data of **1**, **2**, and **3**; Table S2: Fluorescence data of **1b** and **2c** in polymer films; Table S3: Orientational polarizability (Δ*f*), wavelength of absorption maximum (*λ*_abs_), wavelength of fluorescence maximum (*λ*_F_), wave number of absorption maximum (*ν*_a_), wave number of fluorescence maximum (*ν*_F_), and Stokes shift (*ν*_a_–*ν*_F_) of **1c**; Table S4: Orientational polarizability (Δ*f*), wavelength of absorption maximum (*λ*_abs_), wavelength of fluorescence maximum (*λ*_F_), wave number of absorption maximum (*ν*_a_), wave number of fluorescence maximum (*ν*_F_), and Stokes shift (*ν*_a_–*ν*_F_) of **2c**.
