# Supplementary material for: Bis(tricyclic) Aromatic Enes That Exhibit Efficient Fluorescence in the Solid State
Source: Molecules. 2024 Nov 14;29(22):5361. doi: 10.3390/molecules29225361 (PMC11596714; doi:10.3390/molecules29225361)
Supplement: Supplementary file 1 [file molecules-29-05361-s001.zip › molecules-3297366-supplementary.pdf]

Supporting Information

Bis(tricyclic) Aromatic Enes That Exhibit Efficient Fluorescence in the Solid State

Masaki Shimizu [1],* Kenta Nishimura [1], Mizuki Mineyama [1], Rin Terao [1], Tsuneaki Sakurai [1], and Hiroshi Sakaguchi [2]

[1] *Faculty of Molecular Chemistry and Engineering, Kyoto Institute of Technology, 1 Hashikami-cho, Matsugasaki, Sakyo-ku, Kyoto 606-8585, Japan*

[2] *Institute of Advanced Energy, Kyoto University, Gokasho, Uji 611-0011, Kyoto, Japan*

Table of Contents

Figure S1–S6. ^{1}H and ^{13}C NMR Spectra of 2a, 3a, and 3c S2

Table S1. Fluorescence data of **1**, **2**, and **3**. S5

Table S2. Fluorescence data of **1b** and **2c** in polymer films. S6

Figure S7. Fluorescence spectra of **1b** in polymer film. S6

Figure S8. Fluorescence spectra of **2c** in polymer film**.** S6

Figure S9 Absorption and fluorescence spectra of **1c** in various solvents. Excitation wavelength in fluorescence spectra is 420 nm. S7

Table S3. Orientational polarizability (Δf), wavelength of absorption maximum (λ_{abs}), wavelength of fluorescence maximum (λ_F), wave number of absorption maximum (ν_a), wave number of fluorescence maximum (ν_F), and Stokes shift ($\nu_a - \nu_F$) of **1c**. S7

Figure S10. Lippert–Mataga plots of **1c**. S7

Figure S11 Absorption and fluorescence spectra of **2c** in various solvents. Excitation wavelength in fluorescence spectra is 420 nm. S8

Table S4. Orientational polarizability (Δf), wavelength of absorption maximum (λ_{abs}), wavelength of fluorescence maximum (λ_F), wave number of absorption maximum (ν_a), wave number of fluorescence maximum (ν_F), and Stokes shift ($\nu_a - \nu_F$) of **2c**. S8

Figure S12. Lippert–Mataga plots of **2c**. S8

^{1}H and ^{13}C NMR spectra of 2a, 3a, and 3c

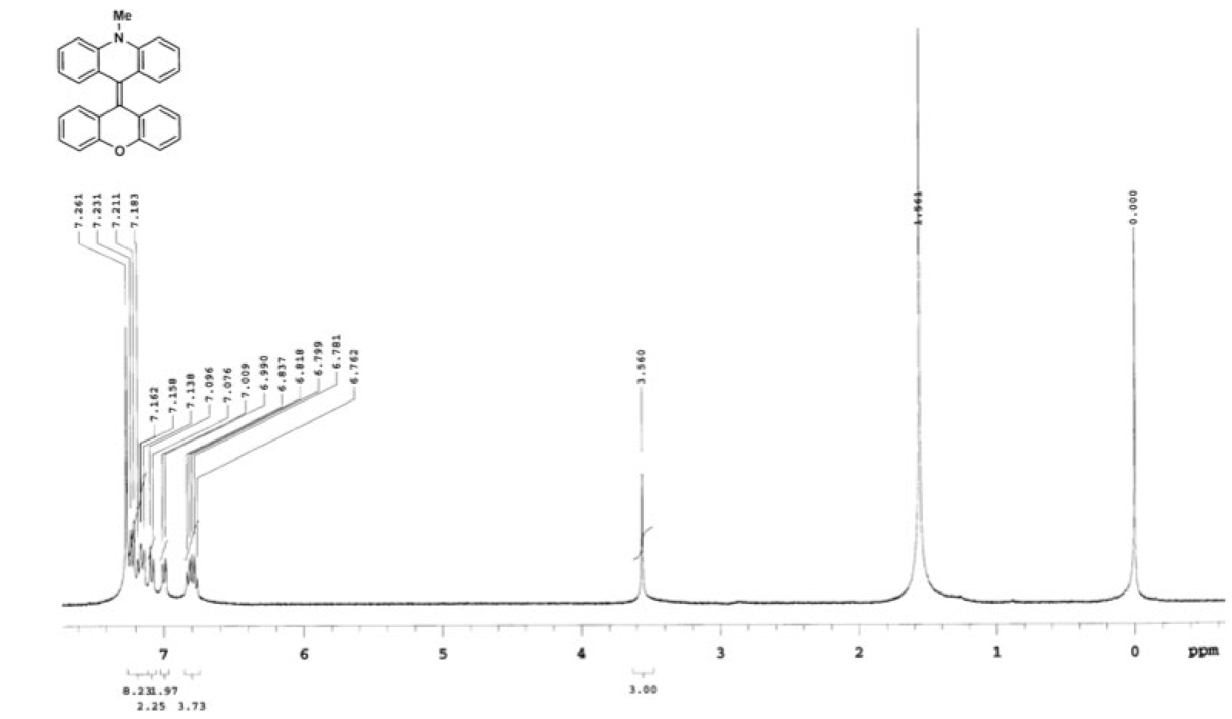

Figure S1. ^{1}H NMR of **2a**.

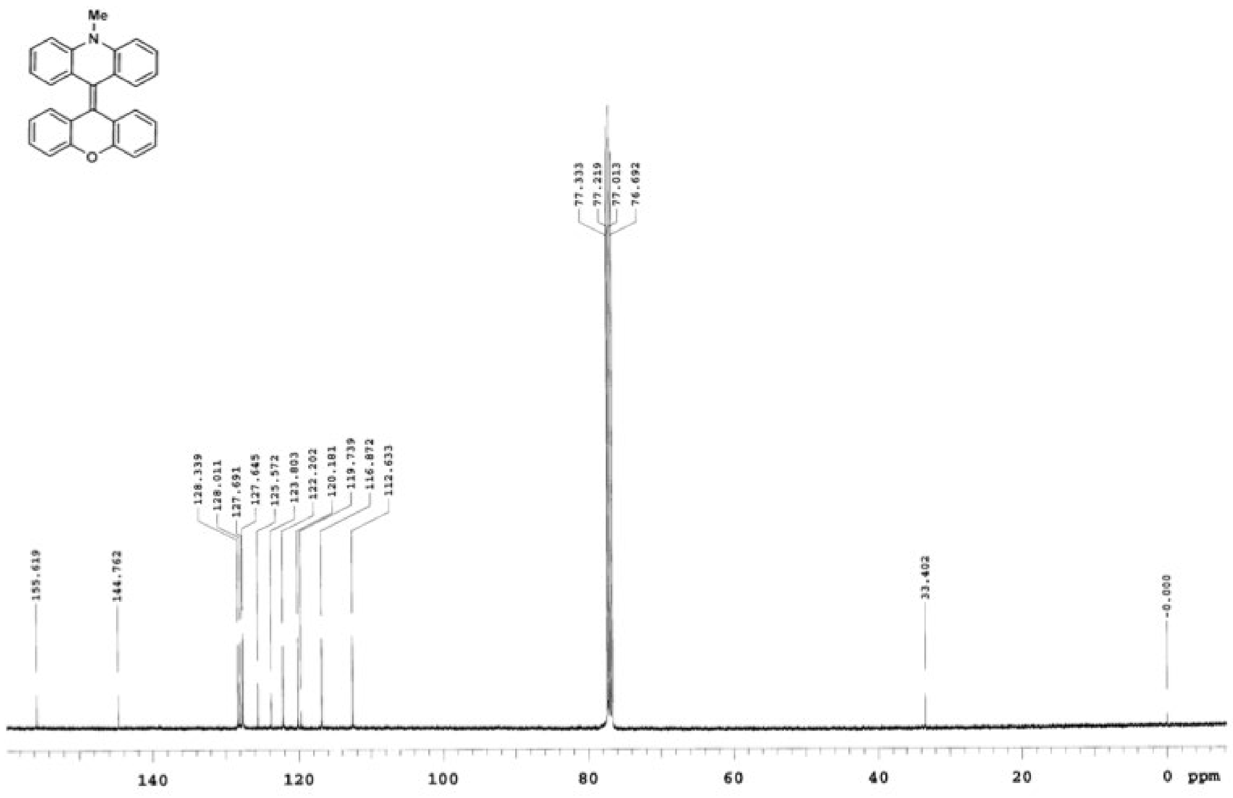

Figure S2. ^{13}C NMR of **2a**.

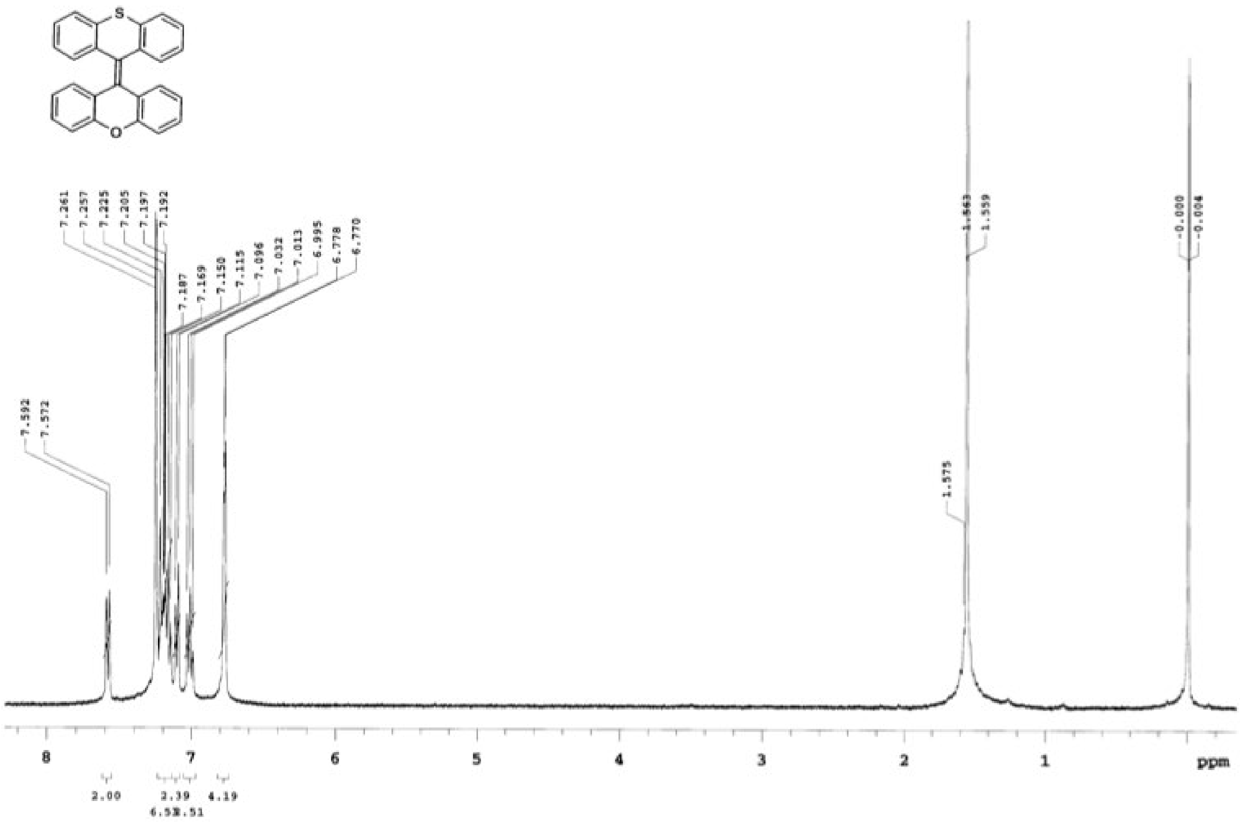

Figure S3. ^{1}H NMR of **3a**.

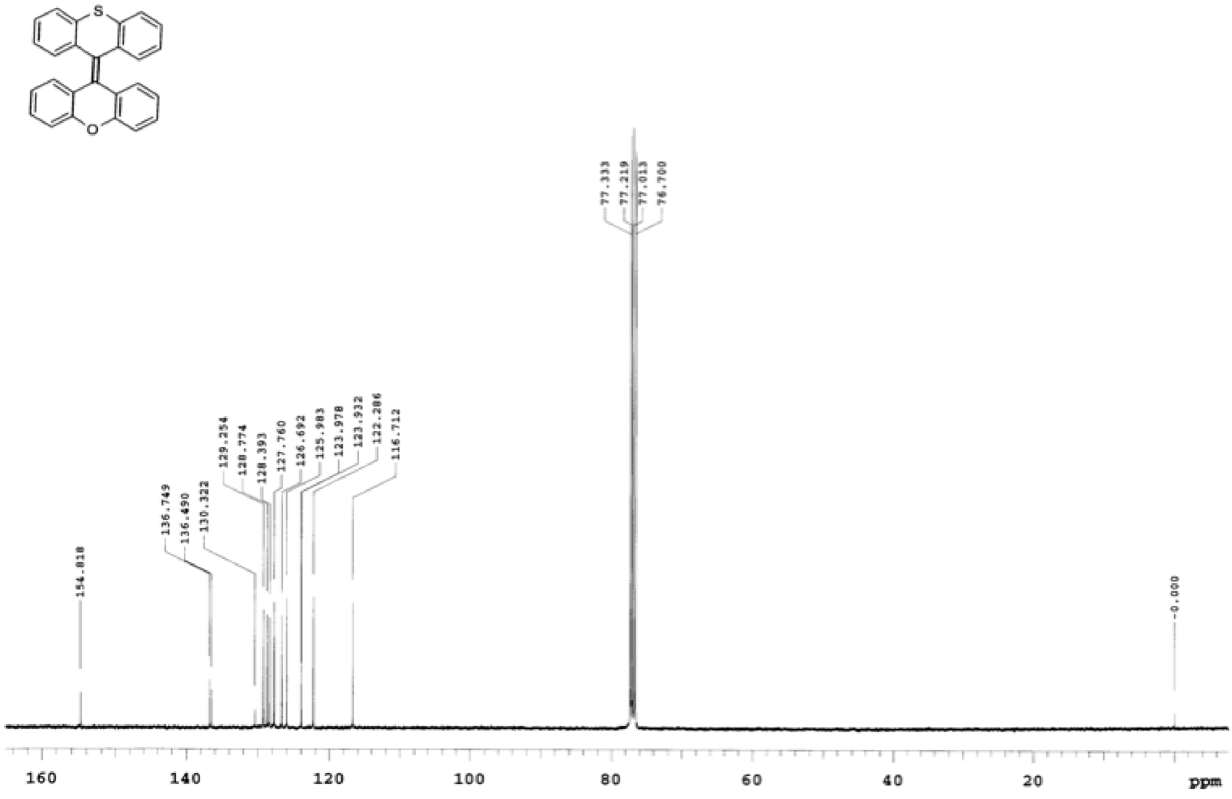

Figure S4. ^{13}C NMR of **3a**.

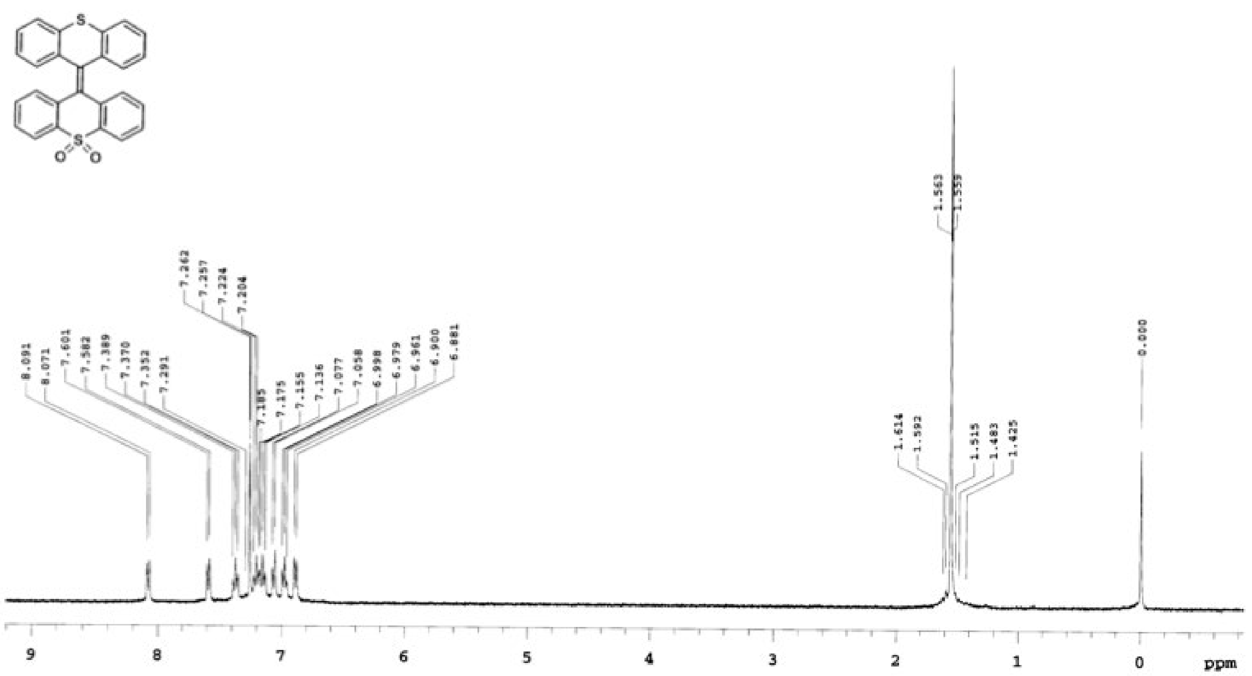

Figure S5. ^{1}H NMR of **3c**.

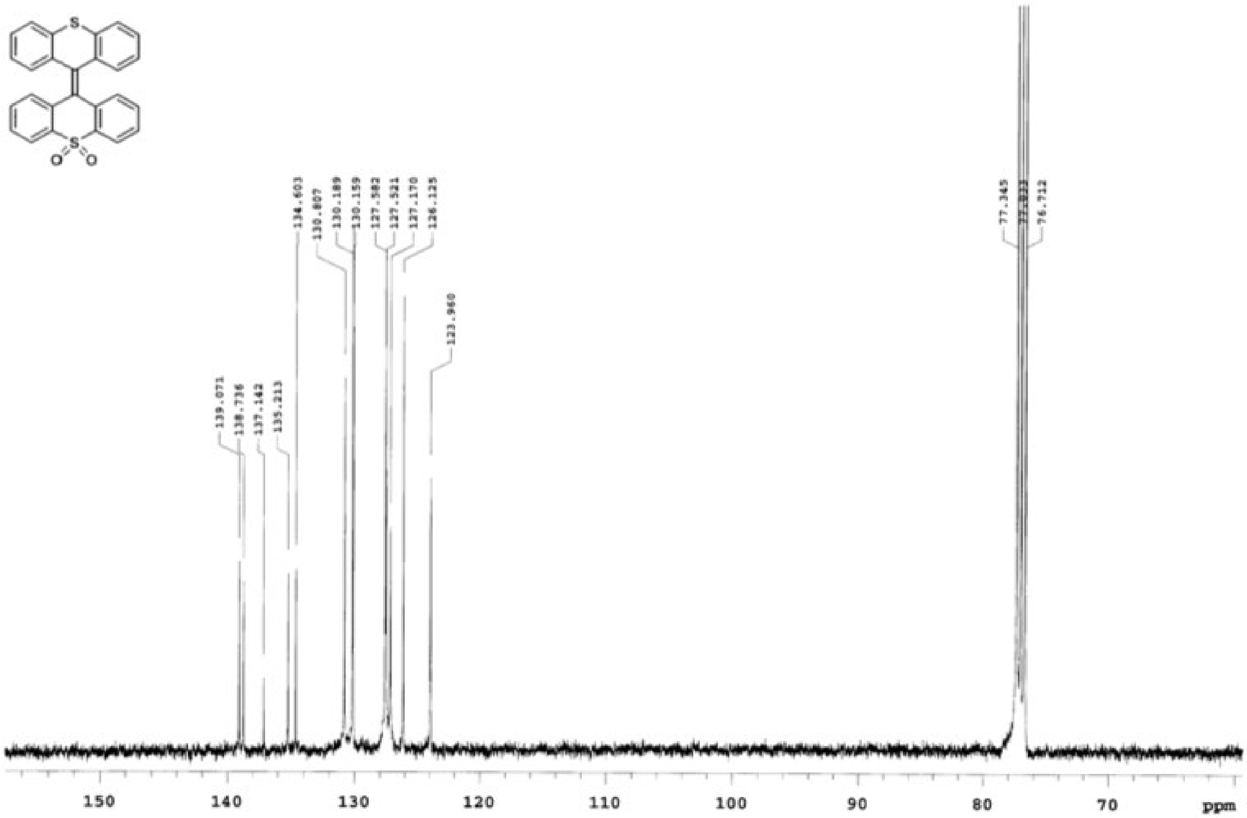

Figure S6. ^{13}C NMR of **3c**.

Fluorescence data including radiative rate constants and nonradiative rate constants of 1, 2, and 3

Table S1. Fluorescence data of **1**, **2**, and **3**.

**1–3**	In toluene[a]					In the solid state[b]					In PMMA[c]				
	λ_{em} [d]	Φ [e]	τ [f]	k_f [g]	k_{nr} [h]	λ_{em} [d]	Φ [e]	τ [f]	k_f [g]	k_{nr} [h]	λ_{em} [d]	Φ [e]	τ [f]	k_f [g]	k_{nr} [h]
**1a**	475	0.20	1.3	1.54	6.15	520	0.81	5.92	1.37	0.32	478	0.81	5.49	1.48	0.35
**1b**	476	0.32	1.9	1.68	3.58	494	0.67	4.42	1.52	0.75	476	0.91	5.56	1.64	0.16
**1c**	493	0.04	0.3	1.33	32.0	513	0.76	4.95	1.54	0.48	496	0.82	4.87	1.68	0.37
**2a**	478	0.37	2.4	1.54	2.63	519	0.45	6.22	0.72	0.88	484	0.84	6.52	1.29	0.25
**2b**	478	0.48	3.2	1.50	1.63	568	0.30	6.49	0.46	1.08	478	0.91	5.98	1.52	0.15
**2c**	494	0.06	0.4	1.50	23.5	521	0.67	5.34	1.25	0.62	506	0.80	5.95	1.34	0.34
**3a**	435	0.32	1.8	1.78	3.78	443	0.88	4.66	1.89	0.26	429	0.67	4.02	1.67	0.82
**3c**	441	0.40	2.2	1.82	2.73	448	0.60	3.54	1.69	1.13	437	0.59	4.13	1.43	0.99

[a] Excitation wavelengths [nm]: 405 for **1a** and **2a**, 396 for **1b** and **2b**, 420 for **1c** and **2c**, 355 for **3a**, and 325 for **3c**. [b] Powder: **1a**, **1b**, **2b**, **2c**; microcrystalline solid: **1c**, **2a**, **3a**, **3c**. Excitation wavelength [nm]: 405 for **1a**, 396 for **1b**, 420 for **1c**, 405 for **2a**, 396 for **2b**, 420 for **2c**, 355 for **3a**, and 325 for **3c**. [c] Excitation wavelength [nm]: 405 for **1a**, 396 for **1b**, 380 for **1c**, 405 for **2a**, 396 for **2b**, 420 for **2c**, 355 for **3a**, and 325 for **3c**. [d] λ_{em}: Fluorescence maximum [nm]. [e] Φ: Fluorescence quantum yield. [f] τ: Fluorescence lifetime [ns]. [g] k_f: Radiative rate constant [10^8 s^{-1}]. [h] k_{nr}: Nonradiative rate constant [10^8 s^{-1}].

Fluorescence data and spectra of 1b and 2c in polymer film

Film Preparation: Poly(methyl methacrylate) (PMMA) was purchased from Wako. Polystyrene (PS), poly(bisphenol A carbonate) (PC), and poly(vinyl acetate) (PVAc) were purchased from Sigma-Aldrich. Each polymer (200 mg) and **1b** or **2c** (0.2 mg) were dissolved in 2 mL of toluene (for **1b**) or $CHCl_3$ (for **2c**), respectively, at 60 °C. The solutions were spin-coated on a quartz plate at 1500 rpm to prepare thin films, which were dried under vacuum before measurement.

As shown in Table S2, the highest fluorescence quantum yields were observed when the BAEs were dispersed in PMMA. The fluorescence spectra were almost the same regardless of the host polymers.

Table S2. Fluorescence data of **1b** and **2c** in polymer films.

	Fluorescence maxima in polymer film[a] (fluorescence quantum yield)			
Host polymer	PMMA	PS	PC	PVAc
**1b**	478 (0.91)	478 (0.87)	479 (0.83)[b]	476 (0.89)
**2c**	504 (0.80)	503 (0.73)	505 (0.71)	506 (0.74)

[a] Absolute quantum yields were recorded using a calibrated integrating sphere. [b] The doped film was prepared from a THF solution of **1b** and PC due to the low solubility of PC in toluene.

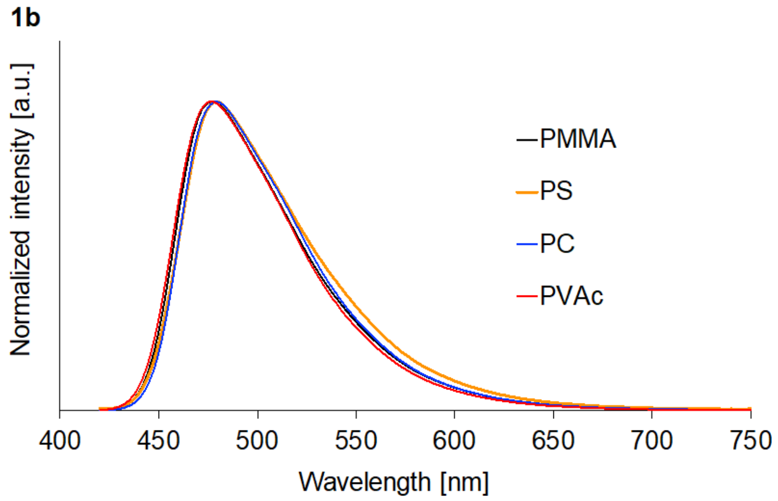

Figure S7. Fluorescence spectra of **1b** in polymer film.

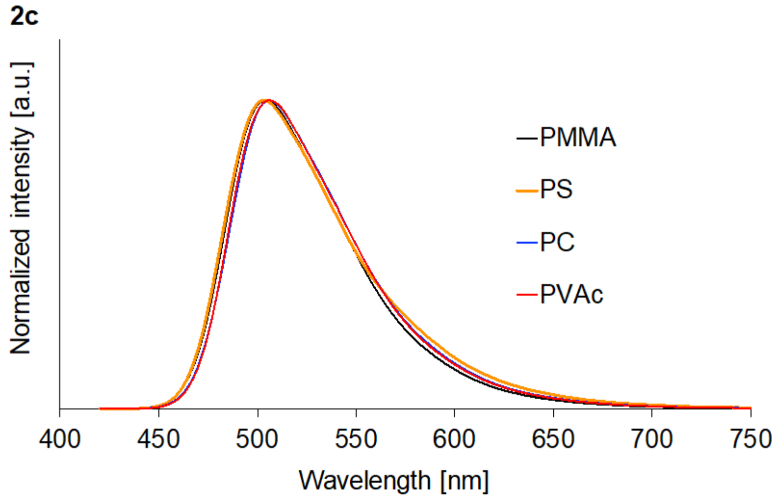

Figure S8. Fluorescence spectra of **2c** in polymer film.

Lippert–Mataga Study on 1c and 2c

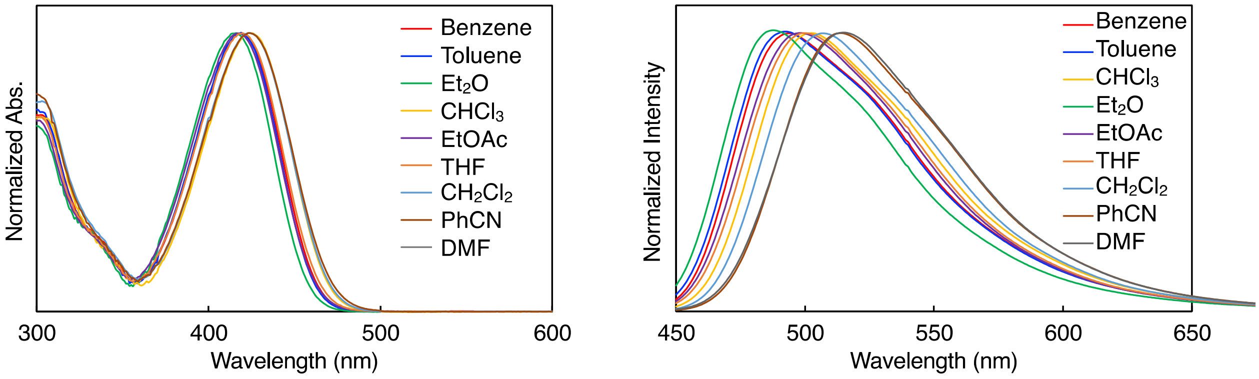

Figure S9. Absorption and fluorescence spectra of **1c** in various solvents. Excitation wavelength in fluorescence spectra is 420 nm.

Table S3. Orientational polarizability (Δf), wavelength of absorption maximum (λ_{abs}), wavelength of fluorescence maximum (λ_F), wave number of absorption maximum (ν_a), wave number of fluorescence maximum (ν_F), and Stokes shift ($\nu_a - \nu_F$) of **1c**.

solvent	Δf^{a}	λ_{abs} (nm)	λ_F (nm)	ν_a (cm^{-1})	ν_F (cm^{-1})	$\nu_a - \nu_F$ (cm^{-1})
benzene	0.003	419.0	494.0	2.39×10^4	2.02×10^4	3.62×10^3
toluene	0.013	419.0	492.0	2.39×10^4	2.03×10^4	3.54×10^3
$CHCl_3$	0.149	424.0	503.0	2.36×10^4	1.99×10^4	3.70×10^3
Et_2O	0.167	415.0	488.0	2.41×10^4	2.05×10^4	3.60×10^3
EtOAc	0.200	417.0	498.0	2.40×10^4	2.01×10^4	3.90×10^3
THF	0.210	419.0	500.0	2.39×10^4	2.00×10^4	3.87×10^3
CH_2Cl_2	0.219	424.0	506.0	2.36×10^4	1.98×10^4	3.82×10^3
PhCN	0.234	427.0	427.0	2.34×10^4	1.95×10^4	3.93×10^3
DMF	0.275	424.0	515.0	2.36×10^4	1.94×10^4	4.17×10^3

a) The values of orientational polarizability (Δf) are cited from Table 7.2 in page 211 of a book "Molecular Fluorescence, B. Valeur, Wiley–VCH, Weinheim, 2002" and Table S3 in B. Yang, et al. *Adv. Funct. Mater.* **2019**, *29*, 1901895.

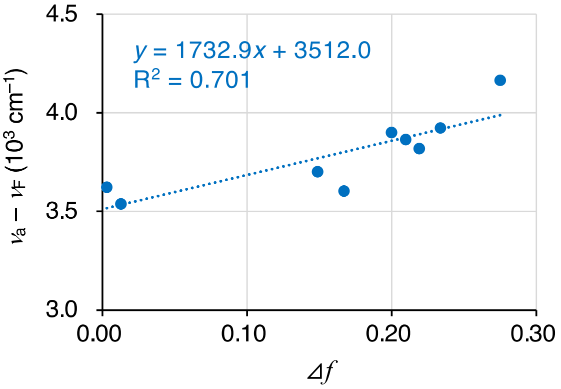

Figure S10. Lippert–Mataga plots of **1c**.

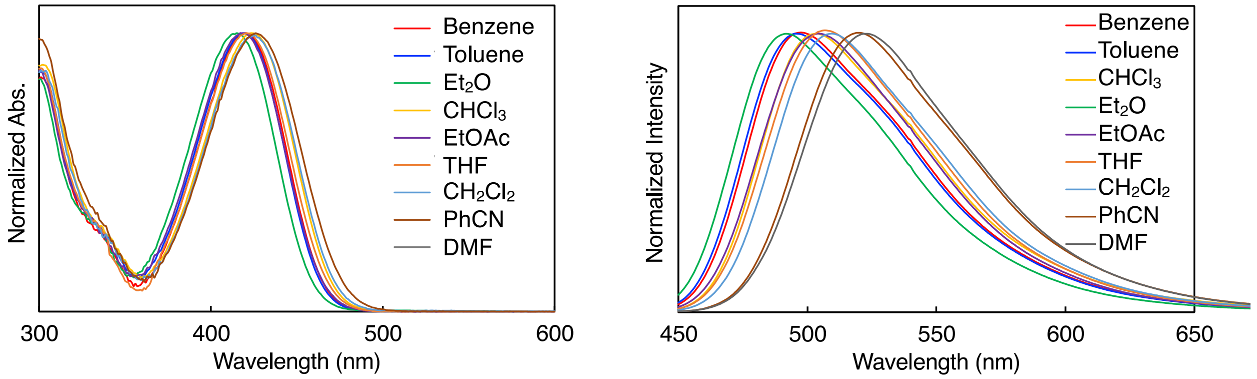

Figure S11. Absorption and fluorescence spectra of **2c** in various solvents. Excitation wavelength in fluorescence spectra is 420 nm.

Table S4. Orientational polarizability (Δf), wavelength of absorption maximum (λ_{abs}), wavelength of fluorescence maximum (λ_F), wave number of absorption maximum (ν_a), wave number of fluorescence maximum (ν_F), and Stokes shift ($\nu_a - \nu_F$) of **2c**.

solvent	Δf^a	λ_{abs} (nm)	λ_F (nm)	ν_a (cm^{-1})	ν_F (cm^{-1})	$\nu_a - \nu_F$ (cm^{-1})
benzene	0.003	420.0	497.0	2.38×10^4	2.01×10^4	3.69×10^3
toluene	0.013	419.0	495.0	2.39×10^4	2.02×10^4	3.66×10^3
$CHCl_3$	0.149	424.0	504.0	2.36×10^4	1.98×10^4	3.74×10^3
Et_2O	0.167	415.0	492.0	2.41×10^4	2.03×10^4	3.77×10^3
EtOAc	0.200	419.0	504.0	2.39×10^4	1.98×10^4	4.03×10^3
THF	0.210	420.0	506.0	2.38×10^4	1.98×10^4	4.05×10^3
CH_2Cl_2	0.219	425.0	509.0	2.35×10^4	1.96×10^4	3.88×10^3
PhCN	0.234	428.5	520.0	2.33×10^4	1.92×10^4	4.11×10^3
DMF	0.275	427.0	522.0	2.34×10^4	1.92×10^4	4.26×10^3

a) The values of orientational polarizability (Δf) are cited from Table 7.2 in page 211 of a book "Molecular Fluorescence, B. Valeur, Wiley–VCH, Weinheim, 2002" and Table S3 in B. Yang, et al. *Adv. Funct. Mater.* **2019**, *29*, 1901895.

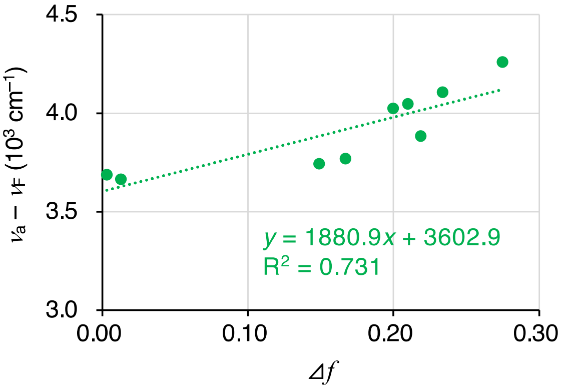

Figure S12. Lippert–Mataga plots of **2c**.
